# Fall risk assessment via multi-batch plantar pressure data fusion: an ensemble framework integrating global and local features

**DOI:** 10.3389/fbioe.2026.1890356

**Published:** 2026-08-14

**Authors:** Siyuan Li, Hongbo Yao, Yue Huang, Zunhao Xu, Ke Qin, Lu Liu, Jingyi Zhang, Qing Zeng, Lin Shu

**Affiliations:** 1 School of Software Engineering, South China University of Technology, Guangzhou, China; 2 School of Future Technology, South China University of Technology, Guangzhou, China; 3 School of Economics and Finance, South China University of Technology, Guangzhou, China; 4 Shien-Ming Wu School of Intelligent Engineering, South China University of Technology, Guangzhou, China; 5 Center of Rehabilitation Medicine, Zhujiang Hospital, Southern Medical University, Guangzhou, China

**Keywords:** ensemble learning, fall risk assessment, global and local features, multi-batch data fusion, plantar pressure, wearable insoles

## Abstract

**Introduction:**

Falls seriously threaten the health and independence of older adults. Existing plantar pressure‐based fall risk assessment methods often rely on single datasets and global features, limiting generalizability across data distributions.

**Methods:**

To enhance cross-batch robustness, this study integrated plantar pressure data from two independent batches with inherent distributional differences. Eighty older adults performed a 2‐min walking task while pressure signals were collected using an intelligent footwear system. A feature set of 44 global and 456 local features was constructed, and a four‐model ensemble comprising two MLPs and two 1D‐CNNs was developed. Model performance was evaluated using 8‐fold stratified cross‐validation, Mann–Whitney U‐based pre‐selection, and SHAP/Grad‐CAM interpretability analyses.

**Results:**

The ensemble achieved an overall accuracy of 86.25%, outperforming the best single model (MLP1, 80.0%). Performance remained stable across the two batches, with accuracies of 86.0% and 86.7%, corresponding to a fluctuation of 0.7 percentage points. This fluctuation was substantially lower than those observed for the single models; for example, CNN2 fluctuated by 9.3 percentage points.

**Discussion:**

These findings suggest that fusing multi‐batch plantar pressure data with an ensemble integrating global and local features can help mitigate the impact of cross-batch distribution shifts on model performance and improve the stability of fall risk assessment, thereby providing a methodological basis for its translation from research settings to practical applications.

## 1 Introduction

Falls are among the most common and most serious adverse events affecting older adults. Epidemiological studies indicate that approximately 30% of individuals aged 65 years and older experience at least one fall each year (Salari et al., 2022; Appeadu and Bordoni, 2026), and the incidence as well as related adverse outcomes—such as hip fractures, intracranial injuries, and long-term functional impairment—increase significantly with advancing age (Fu et al., 2021; Jeon et al., 2025). Falls not only directly compromise the quality of life and independence of older adults, but also impose a long-term burden on healthcare systems and social resources. Therefore, achieving early identification and continuous monitoring of fall risk represents a critical challenge in aging societies. Traditional fall risk assessment methods primarily rely on clinical functional scales, such as the Berg Balance Scale (BBS) and the Timed Up and Go Test (TUGT) (Berg et al., 1992; Shumway-Cook et al., 2000; Lee et al., 2020). Although these approaches are widely used in clinical practice, they are inherently discrete and manually administered, and thus suffer from several limitations, including dependence on trained professionals, subjectivity, and the inability to support long-term continuous monitoring (Friedrich et al., 2021; Miranda-Cantellops and Tiu, 2026). With the rapid development of wearable technologies, sensor-based objective assessment methods have gradually become a major research focus (Wang et al., 2021; Hu et al., 2022; Song et al., 2022; Chiang et al., 2024; Xie et al., 2024; Song et al., 2025; Li et al., 2026). Among sensor-based approaches, plantar pressure insoles provide a wearable and relatively unobtrusive way to capture foot–ground interaction during natural walking. Compared with IMUs, they offer more direct information on regional foot loading and pressure transfer; compared with force platforms, they are more suitable for continuous monitoring outside laboratory settings. Therefore, this study adopts plantar pressure signals as a clinically interpretable wearable modality for fall risk assessment.

Building upon this, numerous recent studies have integrated plantar pressure data with machine learning and deep learning techniques for fall risk assessment or functional balance-related risk classification, obtaining relatively high classification accuracy on individual datasets (Song et al., 2022; Song et al., 2025; Hu et al., 2022; Chiang et al., 2024; Li et al., 2026). However, an important limitation persists: models trained on a single, relatively small dataset often have limited generalizability when applied to data collected under different conditions. This issue has been reported in clinical machine learning, where models trained on a single dataset often exhibit significant performance degradation when evaluated on unseen datasets, even under similar clinical conditions. From a theoretical perspective, this phenomenon is commonly attributed to violations of the independent and identically distributed (IID) assumption (Zhang H. et al., 2021). Existing studies have highlighted that discrepancies between training and testing data distributions can fundamentally undermine model generalization, thereby reducing robustness in real-world applications (Khoee et al., 2024). Such distributional discrepancies are particularly pronounced in wearable sensing and gait analysis (Wu et al., 2023). Existing studies have reported that wearable datasets exhibit substantial variability due to differences in sensor placement, subject populations, and acquisition protocols, which naturally induce distribution shifts across datasets (Palermo et al., 2022). In the context of plantar pressure analysis, these factors can further amplify inter-dataset heterogeneity. Accordingly, models developed on a single and relatively homogeneous dataset may show reduced performance stability when applied to data collected under different conditions. Although previous plantar pressure and wearable-sensing studies have reported promising results, maintaining stable performance under batch-related distributional variation remains an important methodological challenge. Addressing this issue may improve the reliability of plantar pressure-based fall risk assessment and support subsequent validation in broader application scenarios.

Beyond data-related issues, feature modeling strategies also constrain model generalization. Most existing studies rely on a limited set of global statistical features—such as the mean, standard deviation, and trajectory length of the center of pressure (COP)—for modeling (Song et al., 2022; Song et al., 2025). While these features offer a certain degree of physical interpretability, they primarily capture macroscopic gait patterns and fail to represent local details and dynamic variations in plantar pressure distribution. As a result, their discriminative power tends to deteriorate when the data distribution shifts. On the other hand, some studies use deep neural networks to directly learn features from raw data (Hu et al., 2022; Chiang et al., 2024). Although this enhances representational capacity, such models are prone to overfitting under small-sample conditions and often lack cross-subject or cross-batch validation, leaving their true generalization performance uncertain. In addition, certain approaches incorporate demographic information or medical history as auxiliary inputs to improve model performance (Li et al., 2026). However, this strategy is constrained by data availability in real-world applications and undermines the practicality of the system as an independent wearable assessment tool.

To address these limitations, this study proposes a multi-batch fall risk assessment framework that fuses two independent datasets, integrates global and local plantar pressure features, and employs a four-model ensemble consisting of two MLPs and two 1D-CNNs. By exposing the learning process to a broader and more heterogeneous data distribution, the fused design encourages the model to learn discriminative patterns shared across batches rather than patterns specific to a single collection period, thereby supporting cross-batch robustness and practical applicability.

## 2 Materials and methods

### 2.1 Data collection

This study utilizes a merged plantar pressure dataset for fall risk assessment. To improve model generalization under cross-batch conditions, two previously collected independent datasets were integrated. The first dataset (Dataset I in Table 1) comprised 48 older adults from a prior study by Hu et al. (2022), and the second dataset (Dataset II in Table 1) comprised 32 older adults from a prior study by Song et al. (2025). It should be noted that both datasets were originally published as core datasets in those prior studies (Song et al., 2025; Hu et al., 2022). The present study is not a simple replication of those prior analyses; instead, it merges these two independently collected batches with inherent distributional differences for the first time. After merging, a total of 80 older adults were included in the final analysis. For clarity, Table 1 presents the demographic characteristics of each original dataset separately, as well as the combined summary statistics of the merged cohort (Dataset III). Participants in both original datasets were recruited from inpatients in the Department of Rehabilitation, The First Affiliated Hospital of Jinan University. Inclusion criteria required participants to be aged 65 years or older and capable of walking independently for at least two minutes.

**TABLE 1:** Information of datasets.

Subjects	Gender (M/F)	Age (years)	BMI (kg/m^2^)
*Dataset I for fall risk assessment* (Hu et al., 2022)			
24 low fall risk	10/14	72.3 ± 6.0	23.5 ± 2.9
24 high fall risk	15/9	75.9 ± 6.9	23.5 ± 2.8
*Dataset II for fall risk assessment* (Song et al., 2025)			
16 low fall risk	9/7	71.8 ± 5.3	21.7 ± 3.3
16 high fall risk	12/4	75.4 ± 7.1	22.3 ± 4.1
*Dataset III for fall risk assessment (This study)*			
40 low fall risk	19/21	72.1 ± 5.7	22.8 ± 3.2
40 high fall risk	27/13	75.7 ± 6.9	23.0 ± 3.4

No significant differences were observed between the high- and low-risk groups in age, sex, or BMI (p > 0.05 for all).

Prior to data collection, all participants underwent a standardized assessment. Specifically, trained healthcare professionals evaluated participants using the Berg Balance Scale (BBS). In addition, a structured questionnaire was administered to collect relevant information, including sex, age, height, weight, exercise habits, history of falls and rehabilitation, footwear habits, and medication use. All experimental procedures were approved by the Ethics Committee of The First Affiliated Hospital of Jinan University (KY-2020–087) on 24 December 2020. Written informed consent was obtained from all participants prior to participation. Based on the BBS scores, participants were categorized into two groups: those with a total BBS score <40 were classified as the high fall-risk group, while those with a score ≥40 were classified as the low fall-risk group (Muir et al., 2008; Jeon et al., 2017). Therefore, the classification target in this study reflects BBS-defined fall risk associated with functional balance status at the time of assessment.

**FIGURE 1:**
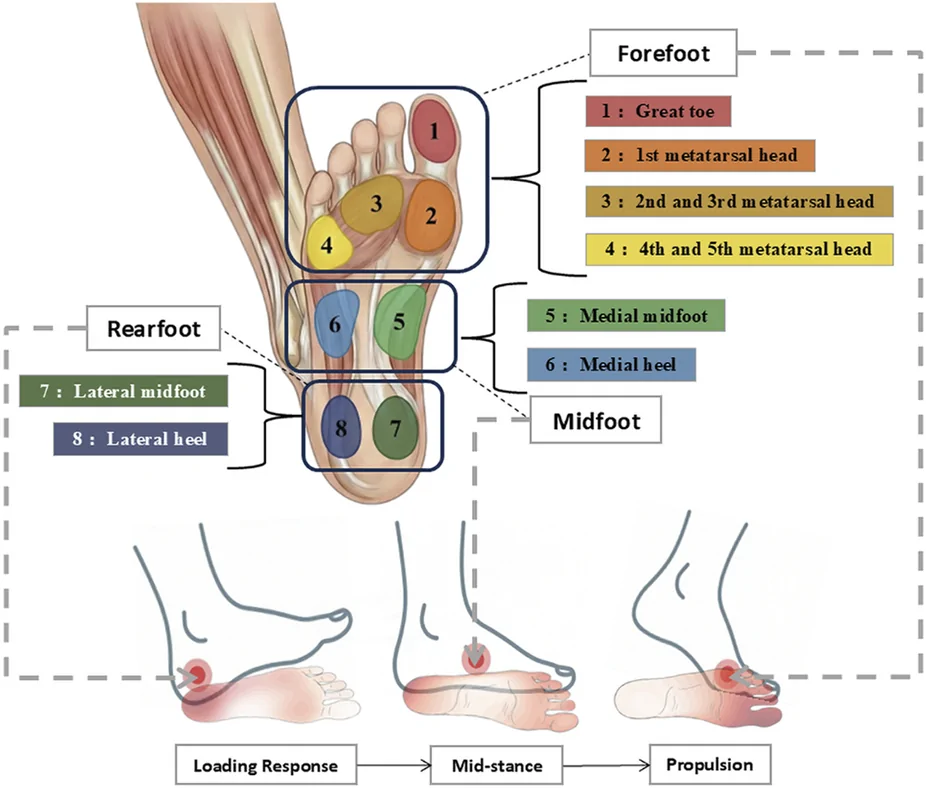
Spatial distribution of plantar sensor regions and their functional roles during the gait cycle.

Plantar pressure data were collected using an intelligent insole system equipped with eight pressure sensors. The spatial distribution of the eight plantar pressure sensors and their functional roles during the gait cycle are illustrated in Figure 1. The system continuously captures pressure signals from different regions of the foot in real time at a sampling frequency of 20 Hz, with data transmitted to a mobile application via Bluetooth. The walking experiment was conducted in a flat corridor of 20 m in length. Participants were instructed to walk at a normal walking speed with a natural gait pattern for more than two minutes under the supervision of healthcare staff to ensure data continuity and reliability. During the experiment, plantar pressure data were continuously recorded, while healthcare professionals simultaneously observed and documented gait characteristics.

It should be noted that the two batches were designed to be theoretically consistent in participant source, walking environment, hardware, and operators. However, several potential sources of batch differences existed during actual collection. The raw data for the first batch were extracted from August 2020 to January 2021, while those for the second batch were extracted from May 2022 to July 2022 – a gap of approximately 1.5 years. This time gap may have introduced slow drift in sensor sensitivity, seasonal variations in laboratory environment (e.g., temperature and humidity), and subtle differences in protocol adherence among operators. Moreover, detailed sensor calibration records were not preserved, so we cannot rule out the effects of device aging or natural inter-individual gait variability. Consequently, although the experimental design was highly consistent, these potential factors could still lead to systematic distributional differences between the two batches. To help readers understand these differences, we present quantitative analyses in Section 3.1 “Cross-Batch Distribution Differences”, where multiple statistical tests reveal the extent of the batch shift and its implications for model generalization.

### 2.2 Gait cycle segmentation

Before feature extraction and construction, the instantaneous total pressure of the left and right feet was first computed from the signals of the eight insole sensors on each side. The resulting pressure signals were smoothed using a 5-sample centered moving-average filter, corresponding to a 0.25-s window at the sampling frequency of 20 Hz. Missing values at the signal boundaries introduced by centered filtering were filled using backward and forward filling. Peak detection was then applied to identify local minima in each foot’s pressure curve, corresponding to toe-off events. Gait cycles for the left foot were defined by two consecutive minima, and within each cycle, a corresponding right-foot minimum was selected as the endpoint to achieve paired segmentation of bilateral gait cycles. To mitigate the effects of gait initiation, the first two steps were discarded, and 90 gait cycles starting from the third step were extracted for subsequent analysis. Across the included gait cycles, the mean gait cycle duration was 1.24 ± 0.20 s.

### 2.3 Global feature extraction

This study adopts a weak-foot-based plantar pressure feature extraction strategy proposed in prior studies. From the segmented gait cycle data, a total of 44-dimensional global features are computed for fall risk assessment (Song et al., 2022; Song et al., 2025). Specifically, the feature set comprises single-foot COP statistical features, symmetry-related features, and temporal consistency-related features. The feature extraction procedure is described as follows.

For each complete gait cycle, the center of pressure (COP) trajectories of the left and right feet are computed separately. The COP coordinates can be calculated using Equation 1 as follows:

$$x_{\text{cop}}=\frac{\sum_{i=1}^{8}F_{i}\cdot x_{i}}{\sum_{i=1}^{8}F_{i}},y_{\text{cop}}=\frac{\sum_{i=1}^{8}F_{i}\cdot y_{i}}{\sum_{i=1}^{8}F_{i}}$$ (1)

where $F_{i}$ denotes the pressure value of the i -th sensor, and $\left(x_{i},y_{i}\right)$ represents the relative coordinates of the sensor in a unified coordinate system. For the COP sequences of the left and right feet, eight fundamental statistical measures are extracted respectively, including mean ( ${\text{mean}}_{x},{\text{mean}}_{y}$ ) and standard deviation ( ${\text{std}}_{x},{\text{std}}_{y}$ ) in the anterior–posterior and medial–lateral directions. Mean resultant distance (MRD) and standard deviation of resultant distance (SRD). Total excursion (TOTEX), defined as the cumulative Euclidean distance of the COP trajectory. 95% confidence circle area (CCA), computed based on the mean and standard deviation of the resultant distance. The “weak foot” side is determined based on the standard deviation of the COP in the anterior–posterior direction: the side with the smaller standard deviation is defined as the weak foot. The weak‐foot criterion is defined in Equation 2 as follows:

$$\text{WeakFoot}=\left\{\begin{array}{cc} \text{Left foot}, & \text{if }{\text{std}}_{y}^{L}\leq {\text{std}}_{y}^{R}\\ \text{Right foot}, & \text{if }{\text{std}}_{y}^{L}>{\text{std}}_{y}^{R} \end{array}\right.$$ (2)

The eight statistical measures of the weak foot are extracted as weak foot features.

Based on the corresponding eight statistical measures of the left and right feet, the gait asymmetry index (GA) is computed using Equation 3 as follows:

$$\text{GA}\left(F_{L},F_{R}\right)=|\ln\left(\frac{\min\left(F_{L},F_{R}\right)}{\max\left(F_{L},F_{R}\right)}\right)|$$ (3)

In addition, spatial distributions of COP points from both feet are used to compute overlap similarity (SIM) and Jensen–Shannon divergence (JSD) to quantify gait symmetry. SIM is defined as the sum of pointwise minimum values between two distributions, while JSD measures the divergence between distributions. The COP sequence of the weak foot is segmented into windows of five consecutive steps. Within each window, the aforementioned eight statistical measures are computed. Then, the standard deviation of these measures across windows (Gait Inconsistency Coefficient, GIC) is calculated, yielding eight gait inconsistency features. Furthermore, sequential similarity (SSIM) and sequential Jensen–Shannon divergence (SJSD) are computed between adjacent windows, resulting in a total of ten temporal consistency-related features.

### 2.4 Construction of the local feature library

To address the limitation of global statistical features in capturing fine-grained gait details under multi-batch scenarios, a plantar pressure local feature library is further constructed. All features are extracted using a two-level structure. In level 1, we compute fundamental indicators within a single gait cycle. In level 2, at the subject level, we compute the mean and standard deviation of these indicators across all gait cycles (90 gait cycles in this study), representing the average gait characteristics and their stability, respectively.

The first category of local features is static pressure features (Yan et al., 2023). Static pressure features are extracted from the raw pressure signals of individual sensors, reflecting the magnitude, distribution, and rate of change of local pressure. Specifically, the following are included:

Peak Pressure (PP), defined in Equation 4, represents the maximum pressure value of a single sensor within a gait cycle:

$$PP_{i}=\underset{t\in \left[0,T\right]}{\max}p_{i}\left(t\right)$$ (4)

where $p_{i}\left(t\right)$ is the pressure of the i -th sensor at time t , and T is the duration of the gait cycle. This metric identifies high-pressure regions of the plantar surface and is used to assess abnormal load distribution.

Pressure-Time Integral (PTI), defined in Equation 5, represents the area under the pressure curve and the total local load:

$$\text{PT}I_{i}=\int_{0}^{T}p_{i}\left(t\right)\text{ dt}\approx \sum_{k=1}^{N-1}\frac{p_{i}\left(k\right)+p_{i}\left(k+1\right)}{2}\cdot \Delta t$$ (5)

where $\Delta t=1/f_{s}$ , and $f_{s}=20\text{ Hz}$ . PTI integrates both pressure magnitude and duration and is a commonly used metric for evaluating total mechanical load on soft tissues.

Average Pressure (AP), defined in Equation 6, represents the arithmetic mean pressure within a gait cycle:

$$AP_{i}=\frac{1}{N}\sum_{k=1}^{N}p_{i}\left(k\right)$$ (6)

which reflects the average loading level experienced by the sensor during the gait cycle.

Pressure Gradient (PG) represents the rate of change of pressure over time. The maximum positive gradient (MaxPG, corresponding to rapid pressure increase) and maximum negative gradient (MinPG, corresponding to rapid pressure decrease) are calculated using Equations 7, 8, respectively:

$${\text{MaxPG}}_{i}=\underset{k}{\max}\frac{p_{i}\left(k+1\right)-p_{i}\left(k\right)}{\Delta t}$$ (7)

$${\text{MinPG}}_{i}=\underset{k}{\min}\frac{p_{i}\left(k+1\right)-p_{i}\left(k\right)}{\Delta t}$$ (8)

Pressure gradients are useful for detecting key gait events such as heel strike and toe-off. Full Width at Half Maximum (FWHM), calculated using Equation 9, represents the duration during which the pressure exceeds half of its peak value:

$$\text{FWH}M_{i}=\left(t_{\text{end}}-t_{\text{start}}\right)\cdot \Delta t$$ (9)

where $t_{\text{start}}$ and $t_{\text{end}}$ denote the first and last time points at which the pressure curve crosses $PP_{i}/2$ , respectively. FWHM characterizes the concentration of pressure distribution: a larger value indicates a more dispersed distribution, while a smaller value indicates a more concentrated pressure.

The second category of local features is temporal features based on stance phase detection. The stance phase is defined as the period within a gait cycle during which the foot is in contact with the ground. Its start and end points are determined using an adaptive thresholding method. Let $t_{\text{start}}$ and $t_{\text{end}}$ denote the onset and offset of the stance phase, respectively. The gait cycle duration is defined as $T_{\text{gait}}=N\cdot \Delta t$ , where N is the number of samples within one gait cycle and Δt is the sampling interval. Based on this, the stance time is given by $T_{\text{supp}}=\left(t_{\text{end}}-t_{\text{start}}\right)\cdot \Delta t$ , and the swing time is defined as $T_{\text{swing}}=T_{\text{gait}}-T_{\text{supp}}$ . The stance phase ratio and swing phase ratio are defined as $R_{\text{supp}}=T_{\text{supp}}/T_{\text{gait}}$ and $R_{\text{swing}}=T_{\text{swing}}/T_{\text{gait}}$ , respectively. Based on these definitions, a set of temporal features are extracted for each gait cycle, as summarized in Supplementary Material Table S1.

To avoid individual bias from fixed thresholds, the optimal threshold for stance phase detection is determined separately for each foot of each subject. Candidate strategies include percentiles (10%, 15%, 20%, 25% of positive pressures), peak-based ratios (5%, 10%, 15%, 20% of peak pressure), standard deviation multiples (0.3, 0.5, 0.7, 1.0 times the pressure standard deviation), mean multiples (0.2, 0.3, 0.4, 0.5 times the average pressure), and hybrids (e.g., the average of the 15% percentile and 10% peak). For each temporal feature, the Mann-Whitney U p-value between high- and low-risk groups and the detection success rate are computed on the training set for each strategy. The strategy with the smallest p-value and a success rate >50% is selected as the optimal threshold for that feature.

The third category of local features is pressure transfer features. These features aim to quantify the dynamic process of load transition from the rearfoot to the forefoot during the stance phase. The forefoot region includes sensors L1–L4 and R1–R4, while the rearfoot region includes L7–L8 and R7–R8. Their total pressures are denoted as $P_{\text{fore}}\left(t\right)$ and $P_{\text{rear}}\left(t\right)$ , respectively. Based on these definitions, the forefoot pressure ratio is defined in Equation 10 as follows:

$$R_{\text{fore}}\left(t\right)=\frac{P_{\text{fore}}\left(t\right)}{P_{\text{fore}}\left(t\right)+P_{\text{rear}}\left(t\right)}$$ (10)

which ranges from 0 (pure rearfoot loading) to 1 (pure forefoot loading). Let $t_{\text{start}}$ and $t_{\text{end}}$ denote the onset and offset of the stance phase, respectively. The peak times of rearfoot and forefoot pressures are denoted as $t_{\text{rear}}$ and $t_{\text{fore}}$ , respectively. The transfer time is defined as $\Delta t_{\text{transfer}}=t_{\text{fore}}-t_{\text{rear}}$ . In addition, $t_{0.25}$ , $t_{0.50}$ , and $t_{0.75}$ represent the first time points at which $R_{\text{fore}}\left(t\right)$ exceeds 0.25, 0.50, and 0.75, respectively. The integral-based features are computed over the stance phase interval $\left[t_{\text{start}},t_{\text{end}}\right]$ . Based on these definitions, a set of pressure transfer features are extracted for each gait cycle, as summarized in Supplementary Material Table S2.

The fourth category of local features is pressure kinetic features. Pressure dynamic features are derived from the total pressure curve $P_{\text{total}}\left(t\right)$ , defined as the sum of all sensor pressures during the stance phase, characterizing the dynamic loading process. The curve typically exhibits a unimodal pattern, consisting of an ascending phase (heel-strike loading) and a descending phase (pre-toe-off unloading). Let $t_{\text{start}}$ and $t_{\text{end}}$ denote the stance phase onset and offset, respectively, and let $t_{\text{peak}}$ represent the time point at which $P_{\text{total}}\left(t\right)$ reaches its maximum value $P_{\text{peak}}$ . The first-order derivative $\frac{dP_{\text{total}}}{\text{dt}}$ describes the instantaneous rate of pressure change. In addition, $x_{i}$ denotes the sampled pressure values within the stance phase, $\overset{ˉ}{x}$ is the mean pressure, and n is the number of samples. Based on these definitions, the pressure kinetic features extracted for each gait cycle are summarized in Supplementary Material Table S3.

The fifth category of local features is load distribution and asymmetry features. These features are designed to quantify the spatial distribution of plantar loads across different anatomical regions of the foot during the stance phase. The load distribution is computed based on the pressure–time integral (PTI), where $\text{PT}I_{i}$ denotes the pressure–time integral of the i -th sensor over the stance phase. The total load is defined as the sum of PTI values across all sensors. The forefoot and rearfoot regions are defined as previously described. In addition, the medial region includes sensors L2, L3, L6, L7, R2, R3, R6, and R7, while the lateral region includes sensors L1, L4, L5, L8, R1, R4, R5, and R8. Based on these definitions, a set of load distribution and asymmetry features are extracted for each gait cycle, as summarized in Supplementary Material Table S4.

To quantify bilateral gait differences, the symmetry index (SI) and gait asymmetry index (GA) are computed for clinically meaningful mean features using Equation 11:

$$SI_{f}=\frac{|f_{L}-f_{R}|}{\frac{1}{2}\left(f_{L}+f_{R}\right)}\times 100,GA_{f}=|f_{L}-f_{R}|$$ (11)

Ultimately, each subject is represented by a local feature vector containing 456 variables. Combined with the previously defined 44-dimensional global features, this forms a complete plantar pressure feature set for fall risk assessment.

### 2.5 Statistical feature selection and ensemble model construction

**FIGURE 2:**
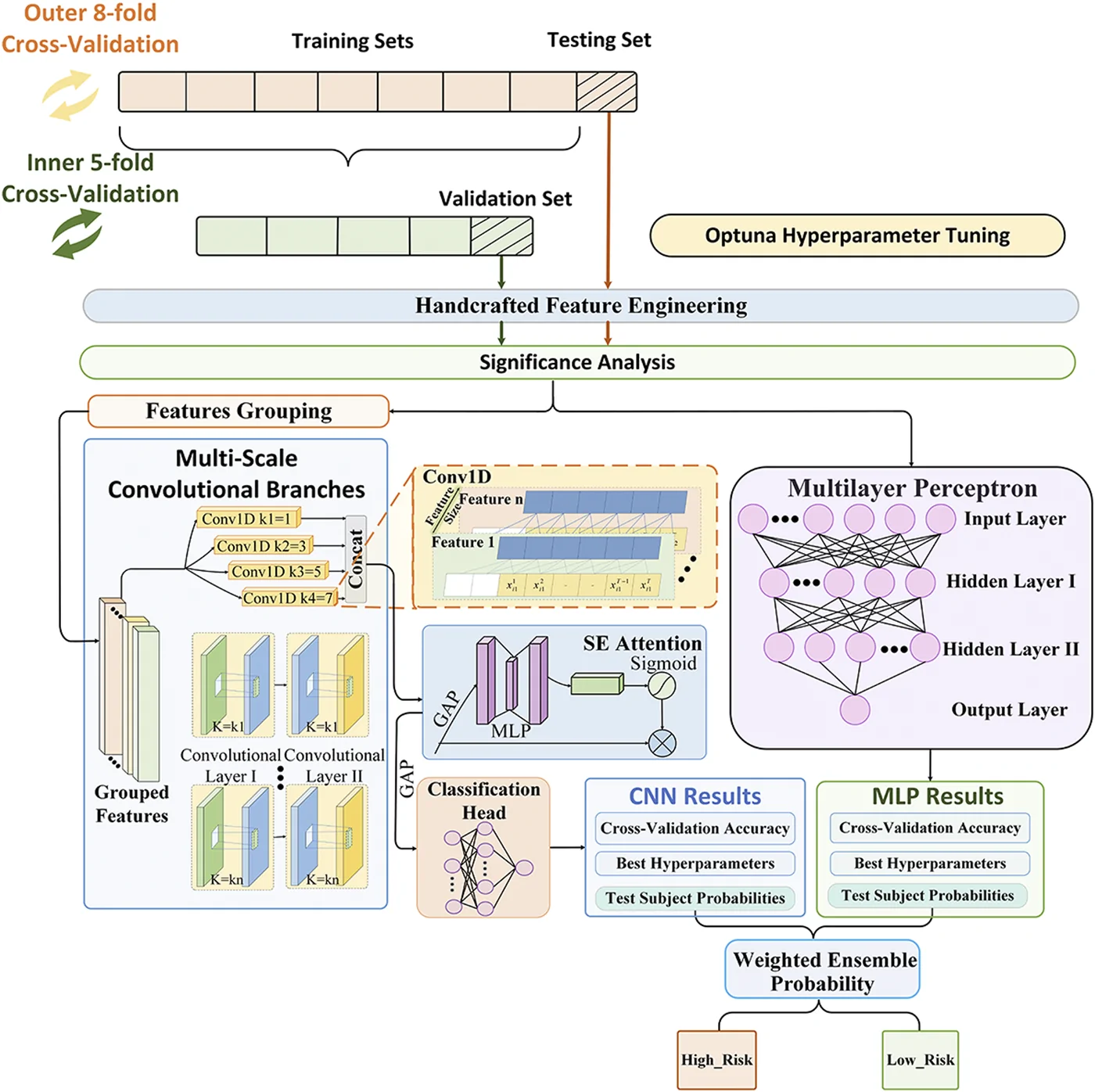
Overview of the statistical analysis and ensemble model construction pipeline.

This study employs four distinct sub-models for ensemble learning: MLP1 (a multilayer perceptron using global features without feature selection), MLP2 (a multilayer perceptron using local features with Mann-Whitney U pre-selection), CNN1 (a multi-scale 1D CNN using global features with anatomically grouped inputs), and CNN2 (a multi-scale 1D CNN using local features with both pre-selection and grouping). These four models extract complementary information from global statistical features and local plantar pressure features, and are finally fused via equal-weight averaging. An overview of the statistical analysis and ensemble model construction pipeline is shown in Figure 2.

To reduce feature redundancy and improve model generalization, the Mann–Whitney U test is employed for feature preselection in MLP2 and CNN2, thereby mitigating the influence of redundant or non-informative local plantar pressure features. Specifically, within each fold of cross-validation, the distributional differences of each feature between the high-risk group (label = 1) and the low-risk group (label = 0) are computed using the training set. Features with P<0.05 are retained for subsequent modeling. In contrast, MLP1 and CNN1 do not perform feature selection and instead utilize all global features directly, ensuring effective extraction from high-dimensional feature representations.

The MLP models (MLP1 and MLP2) adopt fully connected network architectures. Fully connected layers are well-suited for capturing nonlinear combinations among features and are appropriate for structured feature vectors. For global features (MLP1), the network directly leverages relationships among statistical descriptors. For local features (MLP2), the network must learn more complex interactions in a higher-dimensional feature space. The architecture consists of 1–2 hidden layers, with Dropout applied after each layer to control overfitting while maintaining the capacity to learn complex relationships. The CNN models (CNN1 and CNN2) use multi-channel one-dimensional convolutional neural networks. The design rationale is that plantar pressure features exhibit a natural grouping structure (left or right foot, specific anatomical regions, symmetry-related features), and features within the same group are physically correlated. CNNs apply convolutional kernels within each group to capture spatial dependencies among grouped features. To accommodate different “scales” across feature groups, the network incorporates multiple parallel convolutional branches, each with different kernel sizes, enabling the extraction of local patterns at varying receptive fields. The outputs of the branches are adaptively fused via the SE module, allowing the model to automatically focus on channels with greater discriminative power. This design enables flexible utilization of grouped feature information while maintaining sensitivity to multi-scale patterns.

To rigorously evaluate model generalization and mitigate the impact of random data partitioning, we adopt stratified 8-fold cross-validation, where each fold maintains the same proportion of high-risk and low-risk samples as the original dataset. Within each fold, the training set is used for feature selection, hyperparameter optimization, and final model training, while the test set is strictly held out and used only for performance evaluation after training is complete. All evaluation metrics are computed exclusively on the test set. Specifically, for models requiring feature selection (MLP2 and CNN2), a Mann-Whitney U test ( p<0.05 ) is performed independently on the training set of each fold to select features. Hyperparameter optimization is then carried out via nested 5-fold cross-validation within the training set: the Optuna framework is employed to search for the optimal configuration (e.g., network depth, number of neurons, kernel sizes, learning rate) for each model, with the optimization objective being the mean accuracy of the inner 5-fold cross-validation. The search space covers key dimensions including network architecture, regularization strength, and optimizer settings, ensuring that each model is well-adapted to its corresponding feature set and avoiding fixed parameter choices. To improve reproducibility, the final fold-specific architecture and training hyperparameters of the four sub-models, including hidden dimensions, convolutional channels, kernel scales, fully connected units, dropout rate, learning rate, batch size, optimizer, and training epochs, are provided in Supplementary Material Table S9. Finally, the model is retrained on the entire training set using the optimal hyperparameters and evaluated on the test set of the current fold. This nested cross-validation design (outer 8-fold + inner 5-fold) provides a rigorous assessment of generalization performance: the outer 8-fold ensures that performance estimates are averaged over multiple independent test sets, reducing the variance caused by a single arbitrary split; the inner 5-fold prevents information leakage during hyperparameter tuning. Consequently, the reported accuracy is a reliable estimate of how the model would perform on unseen data from the same population.

To integrate the advantages of the four models, an equal-weight averaging ensemble strategy is adopted. During the experimental design, we compared the predictive performance of the four sub-models on the inner validation sets and observed no significant differences. Hence, using simple equal-weight averaging is both reasonable and efficient, while also demonstrating the robustness of our framework across different model outputs. For each test sample, the predicted probabilities from MLP1, MLP2, CNN1, and CNN2 are computed separately, and their arithmetic mean is taken as the final ensemble probability, as shown in Equation 12:

$$P_{\text{ensemble}}=\frac{1}{4}\left(P_{\text{MLP}1}+P_{\text{MLP}2}+P_{\text{CNN}1}+P_{\text{CNN}2}\right)$$ (12)

A sample is classified as high risk (label = 1) when $P_{\text{ensemble}}>0.5$ , otherwise, it is classified as low risk (label = 0). Model performance is evaluated using the mean accuracy (Accuracy) across 8-fold stratified cross-validation. In addition, sensitivity, specificity, positive predictive value (PPV), negative predictive value (NPV), F1 score, and area under the ROC curve (AUC) are also computed. Finally, the prediction results of the test sets from each fold are aggregated, and the predicted probabilities of each individual model as well as the ensemble probabilities are saved for subsequent interpretability analysis.

## 3 Results

### 3.1 Cross-Batch Distribution Differences

To demonstrate distributional differences across datasets, we conducted three experimental analyses. First, we present the batch differences in the raw plantar pressure signals (Supplementary Material Figure S1, 2). From the boxplots, it can be directly observed that for sensors L5 and R5, the first batch exhibits first quartiles (Q1) of both mean pressure and peak pressure that are nearly all greater than the third quartiles (Q3) of the second batch. This anomaly may be related to the generally low and variable loading characteristics of the midfoot region during elderly gait, making these channels more susceptible to experimental disturbances (Hessert et al., 2005; Gimunová et al., 2018). In addition, for sensors L7, L8, R1, R2, R4, R7, and R8, the third quartiles (Q3) of peak pressure in Batch 1 are almost entirely lower than the first quartiles (Q1) of Batch 2. This represents an extremely significant difference, typically reflecting a fundamental change in system state, external conditions, or sensor behavior.

The most pronounced differences (Supplementary Material Tables S5, 6) concentrate in the R1–R3 and L7–L8 regions, where both mean and peak pressures in Batch 2 increase significantly with much higher variability. In contrast, L5 and R5 represent the most significant inverse features, with markedly decreased signal intensity. In particular, L5 may fall into a low-sensitivity, low-noise saturation region. Furthermore, the interquartile ranges (IQR) of peak pressure for L3 and L7 expand considerably, indicating an increase in extreme events. Such pronounced inter-batch differences strongly suggest that the excitation source location, direction, or system operating state in Batch 2 is fundamentally different from that in Batch 1.

To further quantify batch effects in the 44-dimensional global feature space, both an unsupervised multivariate permutation test and a supervised PLS-DA–based permutation test shown in Supplementary Material Figure S3, 4 were conducted. The distance-based permutation test revealed that the observed inter-batch distance (7.489) was substantially greater than the null distribution (1.484 ± 0.398), with a p-value <0.001, indicating statistically significant global distributional differences between the two batches. In addition, the PLS-DA model achieved a high classification accuracy of 0.963. The corresponding permutation test yielded a p-value <0.001, confirming that the observed discriminative performance is significantly better than random chance. Together, these results demonstrate that the two batches exhibit both significant distributional differences and strong separability in the global feature space, suggesting the presence of pronounced batch effects that may impair cross-batch generalization.

Moreover, to evaluate the stability of the discriminative power of global features across datasets, this study compared the significance test results of 44-dimensional global features between the 48-subject dataset (original study) and the 80-subject merged dataset (this study), with the detailed results shown in Supplementary Material Table S7. In the 48-subject dataset, a total of 22 features exhibited significant differences between high-risk and low-risk groups (p < 0.05), among which 9 features reached highly significant levels (p < 0.001), including weak-foot-related features such as MRD^W^, Std_γ_^W^, TOTEX^W^, CCA^W^, CCA^L^, TOTEX^R^, Std_γ_^L^, GIC_Mean_γ_ and TOTEX^L^. These features demonstrated strong discriminative capability in the original study. However, in the 80-subject merged dataset, only 12 features reached statistical significance (p < 0.05), and none achieved p < 0.001. Features that previously exhibited extremely small p-values in the 48-subject dataset (MRD^W^, Std_γ_^W^, TOTEX^W^, CCA^W^, CCA^L^, Std_γ_^L^ and TOTEX^L^) are no longer significant in the merged dataset (p > 0.05). Even among the features that remain significant, their effect sizes are generally small (the effect sizes of GIC_Std_γ_, GIC_Mean_γ_, and similar features are all less than 0.4). These results indicate that as sample size increases and data sources become more heterogeneous, the discriminative power of individual global features decreases substantially, making them insufficient for direct application in cross-batch fall risk identification (Song et al., 2022; Song et al., 2025).

### 3.2 Ensemble model performance evaluation

**FIGURE 3:**
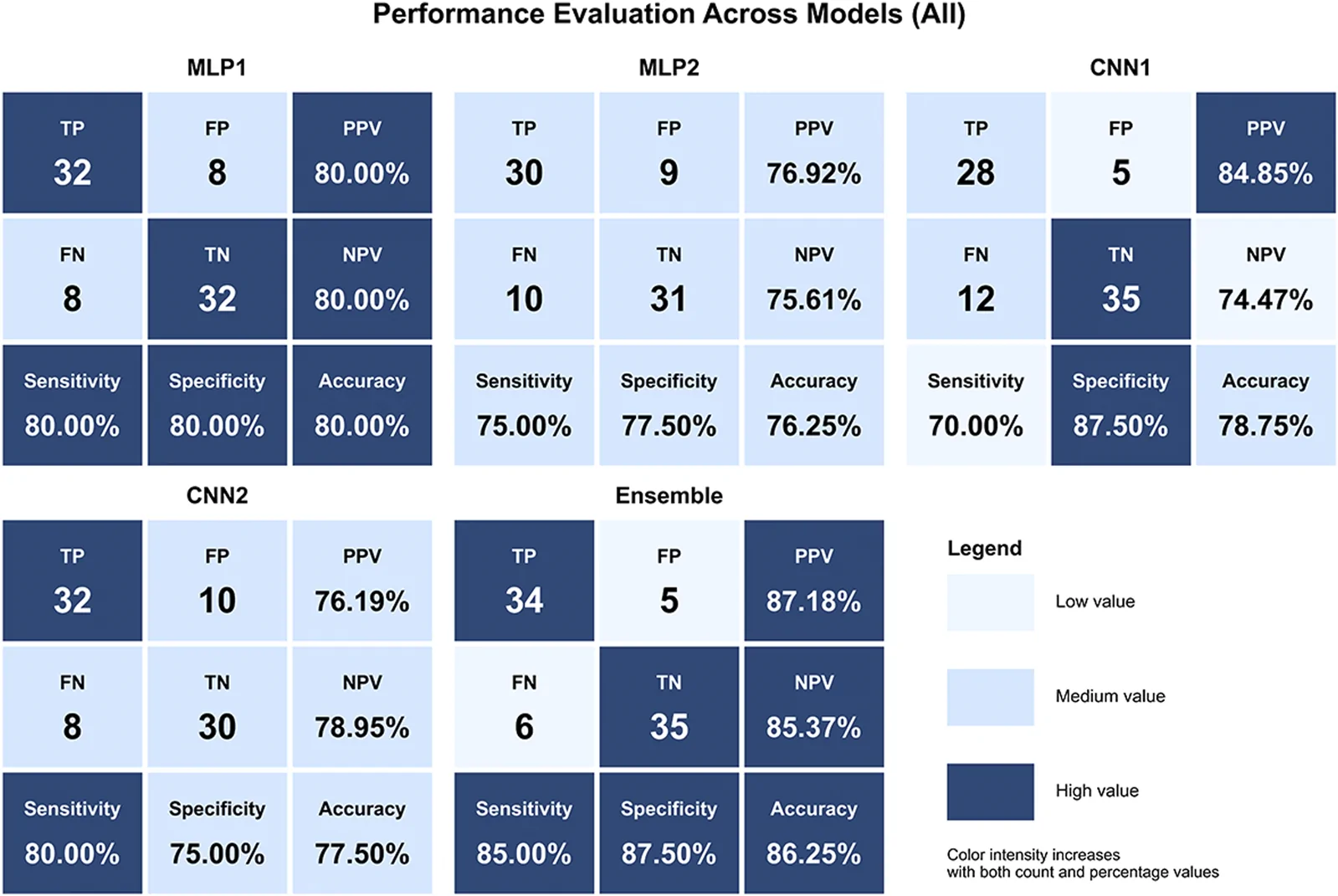
Model performance metrics for the merged dataset (All subjects).

To evaluate the overall performance of the ensemble model, this study computed classification metrics for four individual models (MLP1, MLP2, CNN1, CNN2) as well as the ensemble model on the merged dataset of 80 subjects. The results in Figure 3 show that the ensemble model achieves the best performance across all metrics: an accuracy of 86.25%, sensitivity of 85.0%, specificity of 87.5%, positive predictive value of 87.2%, and negative predictive value of 85.4%. Among the four individual models, MLP1 performs the best (accuracy = 80.0%), followed by CNN1 (78.75%), while CNN2 (77.5%) and MLP2 (76.25%) perform slightly worse. Compared with the best individual model (MLP1), the ensemble model improves accuracy by 6.25 percentage points, sensitivity by 5.0%, and specificity by 7.5%, fully demonstrating the advantages of feature complementarity and architectural complementarity.

To further validate the generalization capability of the ensemble model under cross-distribution scenarios, this study evaluated the performance of each model separately on the two batches, with detailed results presented in Supplementary Material Figures S5, 6. Notably, the ensemble model achieves accuracies of 86.0% and 86.7% on the two batches, respectively, with a difference of only 0.7 percentage points. This is substantially smaller than the performance fluctuations observed in individual models across batches (CNN2 varies from 74.0% to 83.3%, a fluctuation of 9.3 percentage points). This demonstrates that the ensemble strategy effectively mitigates the model’s dependence on specific data distributions and improves cross-batch generalization stability.

The Mann–Whitney U-based pre-selection strategy used in the main experiment was designed as a relatively permissive training-fold-level screening step, rather than as a confirmatory statistical test for individual features. This design aimed to retain sufficient local plantar pressure information, because different local features may represent different biomechanical manifestations of the same clinical phenomenon, such as impaired load transfer, pressure variability, or altered regional support. However, as the number of non-parametric tests increases in a high-dimensional feature space, uncorrected p-value-based screening may increase the risk of false discoveries, and statistical significance alone may not necessarily indicate practical relevance. Therefore, we performed an additional sensitivity analysis using a stricter feature selection criterion. In this sensitivity analysis, Mann–Whitney U-test p-values were adjusted using the Benjamini–Hochberg false discovery rate procedure within each training fold, and rank-biserial effect sizes were calculated. Features were retained only when they satisfied both FDR-adjusted q < 0.05 and |effect size| > 0.20. Under this stricter feature screening setting, the ensemble model achieved an accuracy of 85.00% across 8-fold cross-validation. These results suggest that the proposed ensemble framework maintained robust classification performance even when false discovery control and effect-size filtering were incorporated into the feature pre-selection process.

To further evaluate the stability of the proposed ensemble framework, we additionally performed a repeated-seed stability analysis using 50 random seeds. In this analysis, the 8-fold stratified cross-validation procedure was repeated under different random seeds, while the model configuration selected in the main experiment was kept fixed for computational feasibility. Therefore, this experiment was designed to evaluate the robustness of the selected model configuration under random data partitioning and training stochasticity, rather than to perform repeated hyperparameter optimization for each seed. Across the 50 repeated experiments, the ensemble model achieved a mean accuracy of 81.50%, with a standard deviation of 2.71% and a 95% confidence interval of 80.73%–82.27%. This result provides a conservative estimate of model stability. Considering the small sample size, high-dimensional feature space, and cross-batch heterogeneity in this study, the ensemble framework still maintained an average accuracy above 80% without repeated hyperparameter re-optimization, supporting its robustness under random experimental variation.

To statistically support the model comparisons, we further performed non-parametric tests using the accuracy values obtained from the repeated random-seed experiments. Since all models were evaluated under identical random seeds, the accuracy values were treated as paired repeated measurements. The Friedman test showed a significant overall difference among the five models (Friedman statistic = 120.496, p < 0.001). Pairwise comparisons were then conducted between the ensemble model and each individual model using the Wilcoxon signed-rank test, and the resulting p-values were adjusted using the Benjamini–Hochberg false discovery rate procedure. As shown in Table 2, the ensemble model significantly outperformed all four individual models after FDR correction.

**TABLE 2:** Statistical comparison between the ensemble model and individual models across repeated random-seed experiments.

Comparison	Mean accuracy improvement (% points)	FDR-adjusted p-value
Ensemble vs. MLP1	+2.97	<0.001
Ensemble vs. MLP2	+6.45	<0.001
Ensemble vs. CNN1	+2.20	<0.001
Ensemble vs. CNN2	+5.53	<0.001

Mean improvement was calculated as the paired accuracy difference between the ensemble model and each individual model across repeated random-seed experiments. Pairwise comparisons were performed using the Wilcoxon signed-rank test, and p-values were adjusted using the Benjamini–Hochberg false discovery rate procedure.

### 3.3 Model ablation study

To validate the contribution of each sub-model within the ensemble, four groups of ablation experiments were conducted, in which one model was removed at a time and the performance of the remaining three-model ensemble was evaluated.

**FIGURE 4:**
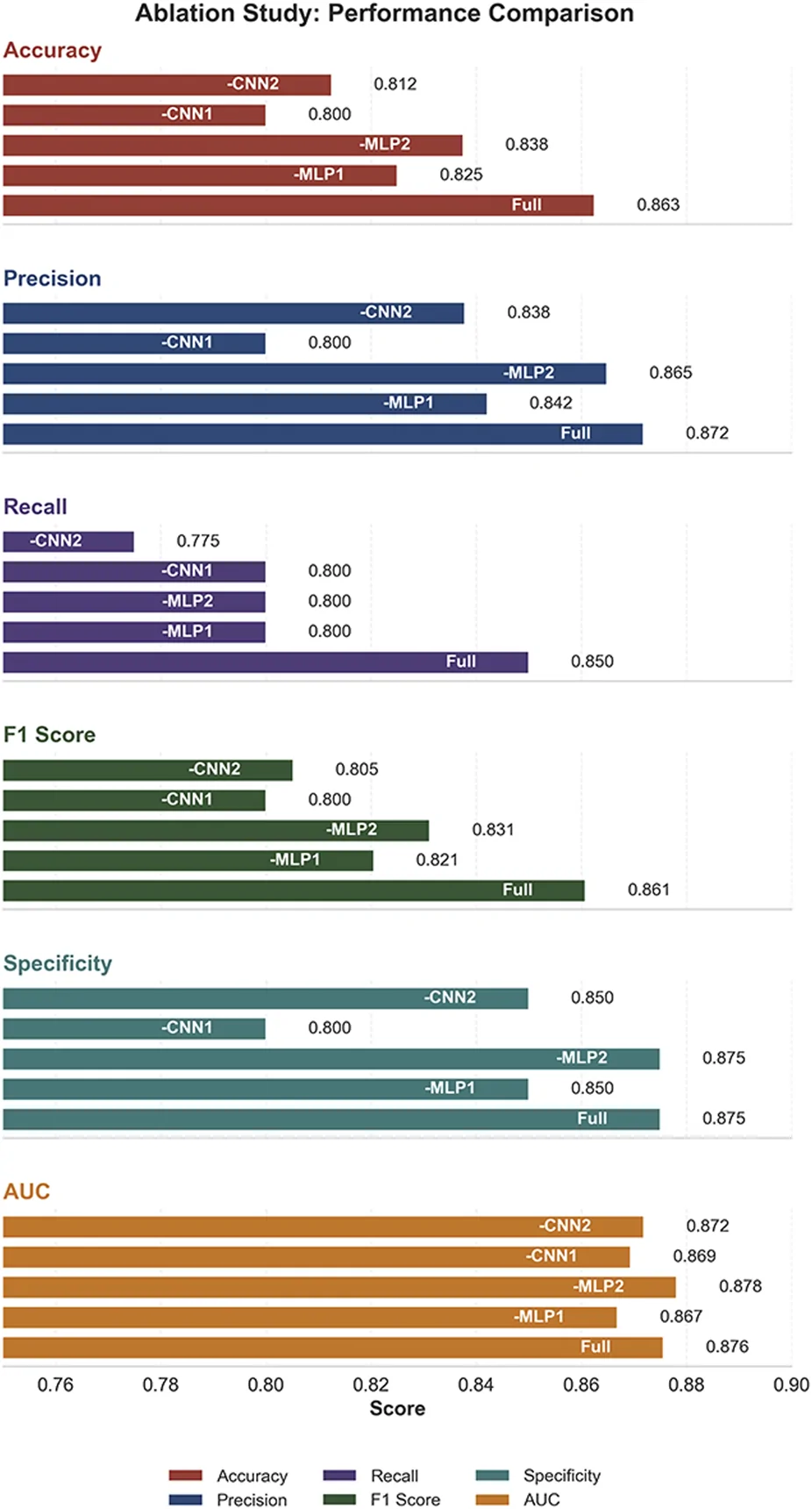
Ablation analysis of the proposed four-model ensemble, showing the performance changes after removing each sub-model across six evaluation metrics.

Ablation analysis in Figure 4 shows that removing any sub-model degrades performance, confirming that all four sub-models are indispensable. Among global feature models, removing CNN1 causes the largest drop: accuracy decreases by 6.25 percentage points and the F1 score falls to 0.800; removing MLP1 reduces accuracy by 3.75 percentage points and recall by 5.0 percentage points. This highlights the critical role of high-dimensional nonlinear global feature combinations and grouped convolution in capturing macroscopic gait abnormalities. For local feature models, removing CNN2 reduces accuracy by 5.0 percentage points, while removing MLP2 reduces it by only 2.5 percentage points, underscoring the advantage of local convolution in capturing micro-level gait abnormalities. Together, these results demonstrate synergistic complementarity between global/local features and between fully connected/convolutional architectures, with the four-model ensemble being necessary for optimal performance.

### 3.4 Feature analysis

**FIGURE 5:**
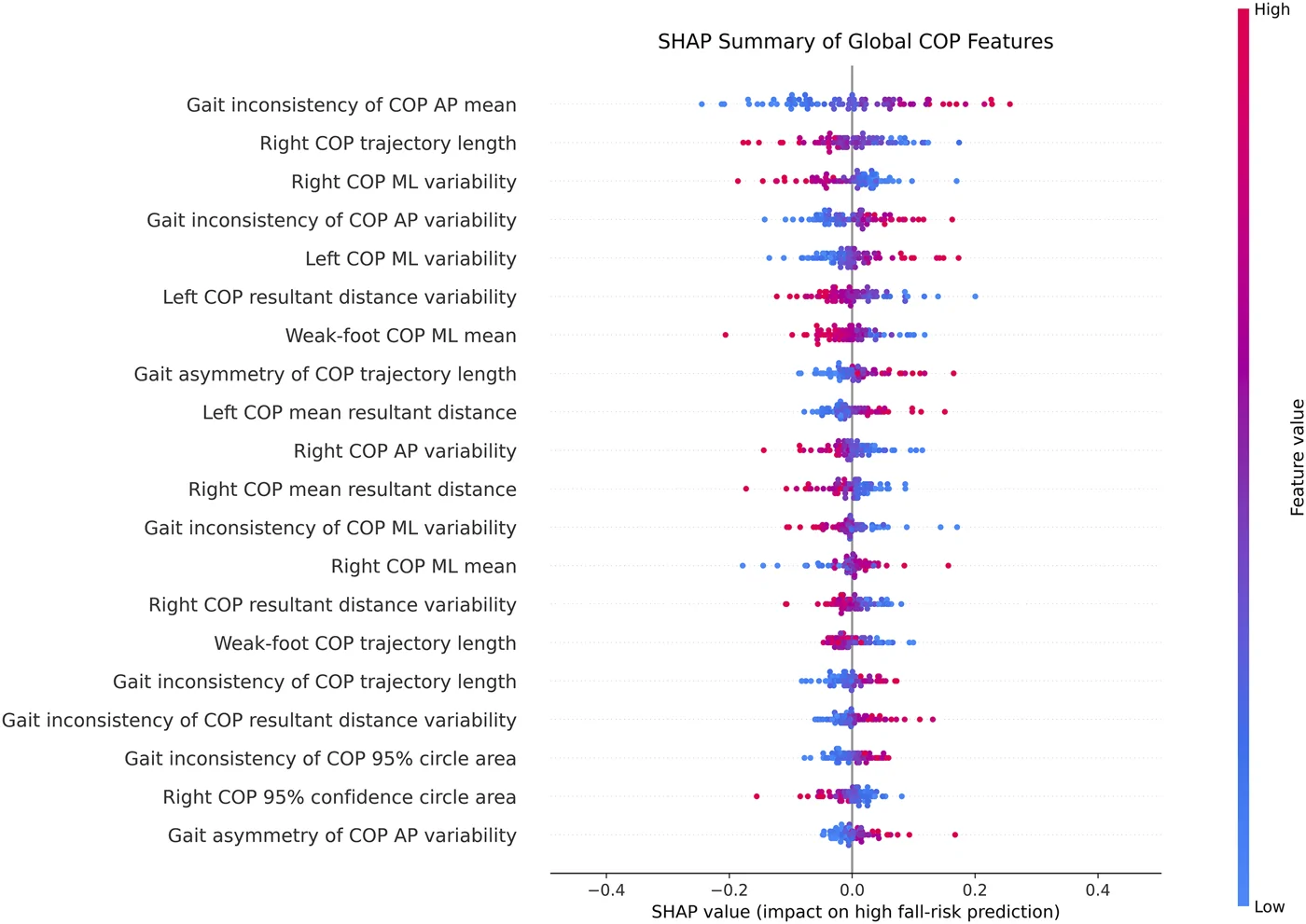
SHAP summary plot for the global feature model (MLP1).

SHAP analysis of MLP1 (Figure 5, see Supplementary Material Table S8 for the mapping of raw feature abbreviations) reveals that the model primarily relies on COP variability metrics (gic_mean_y, gic_std_y) and gait range indicators (totex_R, std_x_R). Higher variability reflects impaired dynamic balance control, while a smaller gait range suggests a restricted and rigid walking pattern. High-risk individuals in our cohort therefore showed a combined pattern of increased instability and reduced gait flexibility, which is consistent with the stiff-but-unstable gait characteristics reported in older fallers (Silva et al., 2024; Terr et al., 2015; Hausdorff et al., 2001; Hausdorff, 2005; Maki, 1997).

**FIGURE 6:**
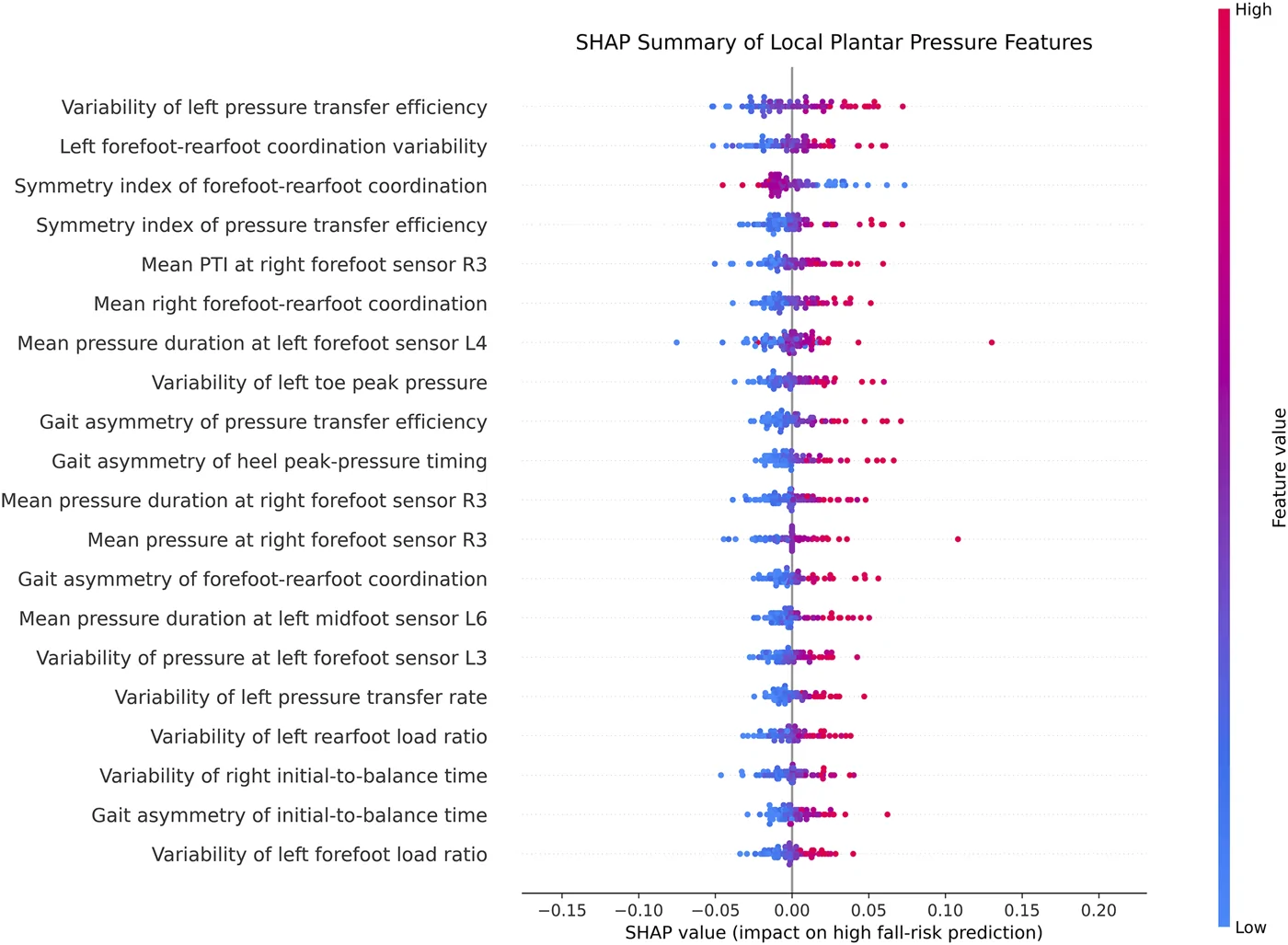
SHAP summary plot for the local feature model (MLP2).

SHAP analysis of MLP2 (Figure 6, see Supplementary Material Table S8 for the mapping of raw feature abbreviations) shows that the top-contributing local features fall into pressure transfer stability, load distribution, and bilateral symmetry indices. Most exhibit a positive association with fall risk, aligning with the understanding that increased plantar pressure variability and impaired load transfer reflect compromised balance (Zhang G. et al., 2021; Kawai et al., 2019; Gerdan et al., 2026; Ou et al., 2026; Orlin and McPoil, 2000). Notably, Fore_Rear_Correlation_Mean_SI shows an inverse relationship (lower values with higher risk). This feature quantifies the consistency of forefoot-rearfoot pressure transfer patterns between feet. A moderate degree of bilateral asymmetry is essential for healthy gait, reflecting natural division of labor between limbs for propulsion and support. When bilateral patterns become highly consistent, it may indicate a generalized decline in forefoot propulsion or reduced gait degrees of freedom, leading to a “loss of functional variability”—a rigid, inflexible gait pattern associated with high fall risk.

Additionally, for each test subject, Grad-CAM was used to generate one-dimensional heatmaps over the feature sequence, and the top five features with the highest activation values (the five most attended positions by the model) were recorded. The frequency with which each feature appeared in the top five across all 80 subjects was then calculated.

Grad-CAM analysis (Table 3, 4) reveals that CNN1 attends most frequently to left-foot COP features (mediolateral sway, trajectory length, and dispersion), also leveraging bilateral COP symmetry information. CNN2 predominantly focuses on variability and width metrics of the left-foot support-propulsion chain, specifically the medial heel (L7), lateral midfoot (L6), and lateral forefoot (L4). These three regions form a continuous chain from heel-strike load absorption to push-off propulsion, and increased variability at these sites indicates an integrated gait dysfunction spanning the entire stance phase (Hu et al., 2024; Lee et al., 2017; Leyh and Feipel, 2022; R et al., 1997; Perry and Burnfield, 2010; Drerup et al., 2008).

**TABLE 3:** CNN1: Features appearing in the top-5 of Grad-CAM importance across test subjects (occurrence count >5).

Feature	Occurrence count	Feature	Occurrence count
std_x_L	32	cca_R	18
totex_L	30	mean_y_L	18
srd_L	30	mean_x_R	15
std_y_L	29	mean_x_L	10
mrd_L	23	cca_L	8

**TABLE 4:** CNN2: Features appearing in the top-5 of Grad-CAM importance across test subjects (occurrence count >5).

Feature	Occurrence count	Feature	Occurrence count
Left_L6_FWHM_Mean	15	Left_L7_PTI_Std	10
Left_L7_AP_Std	13	Left_L3_PTI_Std	9
Left_L4_PTI_Std	12	Left_Balance_Point_Time_Std	9
Left_L4_FWHM_Mean	12	Right_R2_FWHM_Mean	9
Left_L7_FWHM_Mean	11	Left_L6_MinPG_Mean	8

The occurrence count indicates how many times each feature appeared among the top-5, most attended positions (highest Grad-CAM, activation) across all 80 test subjects.

From a clinical gait analysis perspective, we further examined the specific features elevated in the high-risk group. Increased Left_L7_AP_Std and Left_L7_PTI_Std at the medial heel indicate greater variability in heel-contact loading, yet stable heel contact is clinically essential for proper gait initiation. Elevated Left_L6_FWHM_Mean and Left_L6_AP_Std in the lateral midfoot reflect a wider and more fluctuating pressure distribution in the arch area, which clinically often suggests diminished arch support. Higher Left_L4_PTI_Std and Left_L4_FWHM_Mean in the lateral forefoot point to increased variability and reduced efficiency of forefoot propulsion–a typical clinical manifestation of age-related gait changes. Meanwhile, SHAP analysis identifies Left_Transfer_Efficiency_Std (standard deviation of pressure transfer efficiency) as another important predictor; its significantly larger variability in the high-risk group suggests clinically significant impaired functional integration of the entire pressure transfer process from heel to forefoot. This close alignment between the multi-dimensional features and clinical fall mechanisms enhances the credibility of our model’s interpretability.

In addition, the stricter FDR/effect-size sensitivity analysis provided further support for the robustness of the feature interpretation. Under this more conservative screening criterion, the retained or highlighted features were still mainly related to pressure transfer, pressure variability, load distribution, and left-foot indicators. This pattern was broadly consistent with the SHAP and Grad-CAM findings, suggesting that the biomechanical interpretation was not solely driven by the original permissive p-value-based screening strategy.

Notably, both CNN1 and CNN2 consistently assigned higher importance to left-side features across test subjects. This left-sided lateralization may be partially explained by the well-established biomechanical principle of functional division of labor between the lower limbs during walking (Sadeghi et al., 2000). Sadeghi et al. (2000) suggested that lower-limb asymmetry reflects natural functional differences, with the non-dominant limb contributing more to postural stability and stance-phase support, whereas the dominant limb is more involved in propulsion. From this perspective, the higher importance of left-side plantar pressure features may indicate that the left limb played a more stability-related role in the discriminative patterns learned by the models. Empirical findings further support the sensitivity of functionally weaker or stability-demanding limbs to gait deterioration. Si et al. (2024) reported that, among older adults with lower-limb asymmetry, the weaker limb exhibited poorer gait performance. Zhang G. et al. (2021) also found that plantar pressure variability was more pronounced on the side with greater stability demands. These findings are consistent with our observation that left-side plantar pressure features contributed substantially to model discrimination. This functional asymmetry may be reflected in the local features prioritized by CNN2. The three regions with the highest Grad-CAM frequencies—L7 (medial heel), L6 (lateral midfoot), and L4 (lateral forefoot)—form a continuous support–propulsion chain across the stance phase, including heel-strike load absorption, mid-stance lateral arch support, and push-off propulsion (Hu et al., 2024; Lee et al., 2017; Leyh and Feipel, 2022; R et al., 1997; Perry and Burnfield, 2010; Drerup et al., 2008). If the left limb functionally assumed a greater stability-control role in this cohort, its reliance on this chain could make these regions more sensitive to fall-risk-related gait deterioration, such as increased pressure variability and widened pressure distribution. CNN2’s strong attention to variability features across this chain may therefore capture an integrated gait dysfunction spanning from heel strike to toe-off, which may be more detectable on the side with greater stability demands. It should also be emphasized that the “weak foot” defined in the global feature extraction procedure is not equivalent to the preferred foot or dominant foot. In this study, the weak foot was defined algorithmically based on the standard deviation of the COP in the anterior–posterior direction, and therefore represents a data-driven functional asymmetry measure rather than a behavioral foot-preference measure. Therefore, the above interpretation should be treated as a plausible biomechanical hypothesis rather than a confirmed limb-dominance conclusion.

## 4 Discussion

### 4.1 Computational efficiency and potential for real-time implementation

We measured model parameters, training time per fold over 100 epochs, and inference time per sample on two platforms: an NVIDIA RTX 4090 GPU and a 16-vCPU Intel Xeon CPU. For inference, the test set of each fold, containing approximately 10 samples, was processed as a batch. Several warm-up runs were performed before timing, and the final inference time per sample was obtained by averaging over multiple repeated runs.

Table 5 shows that the complete serial ensemble contains 113,268 trainable parameters, indicating a lightweight model structure. The training time per fold was 1–5 s on the GPU and 3–59 s on the CPU. The inference time per sample was 0.55 ms on the GPU and 21.37 ms on the CPU. These results indicate that model inference itself is computationally efficient on both tested platforms. In particular, the CPU inference time was below the 50-ms interval corresponding to the 20 Hz sampling rate, suggesting that the trained model is unlikely to be the computational bottleneck once the required feature vector has been generated. However, deployment on low-power ARM processors may introduce additional latency, and the complete edge implementation, including feature extraction and model inference, should be benchmarked directly in future work. Overall, the proposed framework is computationally lightweight and shows promising feasibility for near-real-time or edge-assisted fall risk assessment.

**TABLE 5:** Computational efficiency of the proposed ensemble framework.

Model	Parameters	Train time (s/fold)	Infer time (ms/sample)
GPU (RTX 4090)			
MLP1	2,718	1.08 ± 0.34	0.049 ± 0.027
MLP2	5,639	0.95 ± 0.46	0.041 ± 0.013
CNN1	45,084	3.48 ± 1.01	0.229 ± 0.052
CNN2	59,827	4.65 ± 0.52	0.230 ± 0.037
Ensemble	113,268	–	0.55 ± 0.09
CPU (16-vCPU xeon)			
MLP1	2,718	3.70 ± 3.37	0.028 ± 0.017
MLP2	5,639	5.38 ± 2.11	0.045 ± 0.010
CNN1	45,084	44.43 ± 13.02	12.26 ± 5.66
CNN2	59,827	58.81 ± 11.28	9.04 ± 2.76
Ensemble	113,268	–	21.37 ± 5.23

### 4.2 Comparison with prior work

As summarized in Table 6, previous studies have several methodological limitations, including reliance on a single dataset, lack of cross-batch validation, and low feature dimensionality, which hinder generalization across different datasets and populations.

**TABLE 6:** Comparison of methodological designs between prior works and this study.

Aspect	Song et al. (2022)	Hu et al. (2022)	Li et al. (2026)	Song et al. (2025)	This study
Data source	Single dataset (48)	Single dataset (48)	Single dataset (48)	Two batches (48 + 32), validated separately	Two batches (80), fused
Validation	Random hold-out	Random split	Random split	Batch-wise testing	Stratified 8-fold CV
Cross-batch evaluation	No	No	No	Yes (but no fusion)	Yes (fusion + CV)
Feature strategy	44 global features	Raw signals + Conv-LSTM	Hybrid WNM + MLP	44 global features	44 global +456 local features
Main limitation	Hard cases need new features	Lack cross-subject validation	Small sample size	Low feature dimensionality	​

Early works (Song et al., 2022; Hu et al., 2022; Li et al., 2026) all relied on a single dataset of 48 subjects with random/fixed partitioning. Despite high accuracy under ideal conditions, they acknowledged limitations: small sample size (Li et al., 2026), lack of cross-subject validation (Hu et al., 2022), and need for new features in hard cases (Song et al., 2022). The common issue is that models trained on a single distribution cannot handle real-world shifts.

Song et al. (2025) then used two independent batches (48 + 32 subjects) with training/testing on each batch separately—a step toward practical applications. However, they noted low feature dimensionality (preventing fine-tuning) and a non-plug-and-play adaptive threshold. More importantly, the two batches were not fused, so the model still learned from a single distribution, missing cross-batch complementarity.

To clarify the technical continuity with our prior work, we retain the core “weak foot” concept from Song et al. (2022) and the local plantar pressure metrics from our cross-sectional study (Yan et al., 2023), while directly adopting the two original datasets from Hu et al. (2022) and Song et al. (2025). Based on these foundations, we make two key extensions: we upgrade the weak-foot features from traditional machine learning classifiers to neural networks (MLP1/CNN1), overcoming the low-dimensionality limitation raised by Song et al. (2025); and we expand the limited local pressure parameters into a systematic 456-dimensional local feature library, answering the call for new features by Song et al. (2022). More importantly, we introduce two novel contributions: cross-batch data fusion–merging two independently collected batches with distinct distributions for the first time, enabling the model to learn from heterogeneous data and directly addressing the sample-size and generalizability concerns of previous single-dataset studies (Song et al., 2022; Song et al., 2025; Hu et al., 2022; Li et al., 2026); and a four-model ensemble framework (MLP1, MLP2, CNN1, CNN2) that integrates global and local features as well as fully-connected and convolutional architectures via equal-weight averaging. This ensemble achieves 86.25% accuracy on the merged dataset and reduces cross-batch performance fluctuation to only 0.7 percentage points (compared to 9.3 pp for a single model, CNN2). These results highlight the value of incorporating batch-related heterogeneity into plantar pressure-based fall risk assessment and provide a useful foundation for subsequent validation across broader clinical and wearable-sensing scenarios.

### 4.3 Limitations

Although this study achieved reasonably robust cross-batch performance through multi-batch data fusion and ensemble learning, several limitations exist. First, the merged dataset included only 80 older inpatients from a single hospital, and plantar pressure data were collected using one specific eight-sensor insole system. Although cross-batch robustness was observed after fusing the two batches, both batches were collected from the same hospital using the same plantar pressure system. Therefore, our findings should be interpreted as cross-batch generalization within a same-center and same-device setting, rather than broader cross-dataset generalization across centers, devices, or populations. Multi-center validation involving larger and more diverse populations and different sensor configurations is required before clinical deployment. Second, fall risk classification was based on the Berg Balance Scale, which reflects functional balance and fall risk at the time of assessment rather than prospectively recorded fall events. Thus, our model assesses BBS-defined fall risk status, not future fall prediction. The present framework was not designed to replace clinical history-taking or established functional scales; instead, it provides an objective plantar pressure-based representation of gait and balance-related biomechanics. Future longitudinal follow-up studies tracking actual fall events are needed to determine whether plantar-pressure features provide incremental predictive value beyond BBS scores, prior fall history, and other routine clinical variables, thereby strengthening the clinical evidence for prospective fall prediction. Third, the handcrafted 456-dimensional local feature library, while improving discriminative ability, may have information redundancy and may not fully capture deep spatiotemporal patterns. Although Mann-Whitney U test (p < 0.05) reduced the number of features to ≈100 per fold, some redundancy may remain given the moderate sample size of 80 subjects. Future work will focus on expanding sample size, multi-center external validation, and prospective fall-tracking studies, as well as adopting correlation analysis, stricter feature selection thresholds (e.g., p < 0.01) or dimensionality reduction methods (PCA, LASSO, autoencoders) to facilitate clinical translation. Fourth, the preferred foot of participants was not determined using standard assessments such as a ball-kick test or a validated footedness questionnaire. Therefore, although the observed left-side feature dominance is consistent with the biomechanical hypothesis that the limb with greater stability-control demands may be more sensitive to fall-risk-related gait deterioration, this pattern cannot be definitively attributed to limb dominance. Future studies should include standardized preferred-foot assessments to directly examine whether limb dominance contributes to side-specific plantar-pressure feature importance. Fifth, although age, sex, and BMI showed no significant differences between the high- and low-risk groups and were not used as model inputs, the inpatient cohort may still have had heterogeneous clinical backgrounds, such as differences in comorbidities, medication use, rehabilitation status, and prior fall history. Owing to the limited sample size, this study could not perform reliable subgroup analyses to examine the influence of these clinical factors. Therefore, the identified plantar-pressure features should be interpreted as biomechanical correlates of BBS-defined functional balance-related fall risk, rather than disease-specific pathophysiological markers.

## 5 Conclusion

This study proposes a four-model ensemble framework that integrates global and local plantar pressure features to improve cross-batch fall risk assessment. By combining complementary feature representations, the framework achieved 86.25% accuracy and maintained stable performance across batches, while ablation analysis verified the contribution of each sub-model. These results demonstrate that leakage-free multi-batch fusion is an effective strategy for enhancing the robustness of plantar pressure-based fall risk assessment. Future work will expand the dataset, conduct external validation, and further optimize the framework for wearable monitoring applications.

## Data Availability

The data analyzed in this study is subject to the following licenses/restrictions: The datasets generated or analyzed during this study are available from the corresponding authors on reasonable request. Requests to access these datasets should be directed to LS, shul@scut.edu.cn.
