# Supplementary material for: Fall risk assessment via multi-batch plantar pressure data fusion: an ensemble framework integrating global and local features
Source: Front Bioeng Biotechnol. 2026 Aug 14;14:1890356. doi: 10.3389/fbioe.2026.1890356 (PMC13522186; doi:10.3389/fbioe.2026.1890356)
Supplement: Supplementary file 1 [file Supplementaryfile1.docx]

Supplementary Information for

**Fall Risk Assessment via Data Fusion of Multi-Batch Plantar Pressure: A Global–Local Feature Integrated Ensemble Framework**

Siyuan Li *et al*.

Corresponding authors: Hongbo Yao: [hongboyao2023@163.com](mailto:hongboyao2023@163.com), Qing Zeng: [zengqingyang203@126.com](mailto:zengqingyang203@126.com), Lin Shu: [shul@scut.edu.cn](mailto:shul@scut.edu.cn),

**This PDF file includes:**

Supplementary Fig. S1 to S6

Supplementary Tables S1 to S9

Table S1. Temporal features based on stance phase detection

| Temporal features | Formula / Description |
| --- | --- |
| Stance time | $T_{supp}=(t_{end}-t_{start})\cdot\Delta t$ |
| Swing time | $T_{swing}=T_{gait}-T_{supp}$ |
| Gait cycle duration | $T_{gait}=N\cdot\Delta t$ |
| Stance phase ratio | $R_{supp}=\frac{T_{supp}}{T_{gait}}$ |
| Swing phase ratio | $R_{swing}=\frac{T_{swing}}{T_{gait}}$ |
| Optimal threshold | Adaptive pressure threshold used for stance phase detection for each subject |
| Stance onset time | $t_{start}$ |
| Stance offset time | $t_{end}$ |

Table S2. Pressure transfer features

| Pressure transfer features | Formula / Description |
| --- | --- |
| Rearfoot peak time | $t_{rear}=arg{max}_{t}P_{rear}(t)$ |
| Forefoot peak time | $t_{fore}=arg{max}_{t}P_{fore}(t)$ |
| Transfer time | $\Delta t_{transfer}=t_{fore}-t_{rear}$ |
| Rearfoot peak pressure | $maxP_{rear}(t)$ |
| Forefoot peak pressure | $maxP_{fore}(t)$ |
| Initial transfer point | $t_{0.25}$: first time $R_{fore}(t)>0.25$ |
| Balance point | $t_{0.50}$: first time $R_{fore}(t)>0.50$ |
| Forefoot dominance point | $t_{0.75}$: first time $R_{fore}(t)>0.75$ |
| Initial-to-balance duration | $\Delta t_{\left( 0.25 - 0.50 \right)}=t_{0.50}-t_{0.25}$ |
| Balance-to-dominance duration | $\Delta t_{\left( 0.50 - 0.75 \right)}=t_{0.75}-t_{0.50}$ |
| Forefoot load integral ratio | $\mathrm{FrontLoad}_{int}=\frac{\int_{t_{start}}^{t_{end}} P_{fore}(t)\text{ }dt}{\int_{t_{start}}^{t_{end}} (P_{fore}(t)+P_{rear}(t))\text{ }dt}$ |
| Rearfoot load integral ratio | $\mathrm{RearLoad}_{int}=1-\mathrm{FrontLoad}_{int}$ |
| Integral load ratio | $\mathrm{Ratio}_{int}=\frac{\mathrm{FrontLoad}_{int}}{\mathrm{RearLoad}_{int}}$ |
| Pressure transfer rate | $\text{Rate}=\mid\frac{maxP_{\text{fore}}-maxP_{\text{rear}}}{\Delta t_{\text{transfer}}}\mid$ |
| Forefoot–rearfoot pressure correlation | Pearson correlation coefficient $r(P_{fore},P_{rear})$ |
| Transfer efficiency | $\eta=1-\frac{\mid\Delta t_{\text{transfer}}\mid}{T_{\text{supp}}}$ |

Table S3. Pressure kinetic features

| Pressure kinetic features | Formula / Description |
| --- | --- |
| Peak pressure | $P_{peak}={max}_{t}P_{total}(t)$ |
| Peak time (relative to stance phase) | $t_{peak}=arg{max}_{t}P_{total}(t)$ |
| Rise time | $T_{rise}=t_{peak}-t_{start}$ |
| Mean rise rate | $\bar{v}_{rise}=\frac{P_{peak}-P_{total}(t_{start})}{T_{rise}}$ |
| Maximum rise rate | $v_{rise,max}={max}_{t\in[t_{start},t_{peak}]}\frac{dP_{total}}{dt}$ |
| Rise phase integral | $\int_{t_{start}}^{t_{peak}} P_{total}(t)\text{ }dt$ |
| Fall time | $T_{fall}=t_{end}-t_{peak}$ |
| Mean fall rate | $\bar{v}_{fall}=\frac{P_{peak}-P_{total}(t_{end})}{T_{fall}}$ |
| Maximum fall rate (absolute value) | $v_{\text{fall,max}}=\underset{t\in[t_{\text{peak}},t_{\text{end}}]}{max}\mid\frac{dP_{\text{total}}}{dt}\mid$ |
| Fall phase integral | $\int_{t_{peak}}^{t_{end}} P_{total}(t)\text{ }dt$ |
| Rise-to-fall ratio | $R_{rise/fall}=\frac{T_{rise}}{T_{fall}}$ |
| Skewness | $\frac{\frac{1}{n}\sum(x_{i}-\bar{x})^{3}}{\left( \frac{1}{n}\sum(x_{i}-\bar{x})^{2} \right)^{3/2}}$ |
| Kurtosis | $\frac{\frac{1}{n}\sum(x_{i}-\bar{x})^{4}}{\left( \frac{1}{n}\sum(x_{i}-\bar{x})^{2} \right)^{2}}-3$ |

Table S4. Load distribution features

| Load distribution features | Formula / Description |
| --- | --- |
| Forefoot load ratio | $ForefootLoad=\frac{\sum_{i\in forefoot} PTI_{i}}{\sum_{i=1}^{8} PTI_{i}}$ |
| Rearfoot load ratio | $RearfootLoad=\frac{\sum_{i\in rearfoot} PTI_{i}}{\sum_{i=1}^{8} PTI_{i}}$ |
| Forefoot-to-rearfoot ratio | $Forefoot/Rearfoot=\frac{\mathrm{ForefootLoad}}{\mathrm{RearfootLoad}}$ |
| Medial load ratio | $MedialLoad=\frac{\sum_{i\in medial} PTI_{i}}{\sum_{i=1}^{8} PTI_{i}}$ |
| Lateral load ratio | $LateralLoad=\frac{\sum_{i\in lateral} PTI_{i}}{\sum_{i=1}^{8} PTI_{i}}$ |
| Medial-to-lateral ratio | $Medial/Lateral=\frac{\mathrm{MedialLoad}}{\mathrm{LateralLoad}}$ |

Table S5. Significant changes in median (|change| ≥ 30%)

| Sensor | Metric | Batch1 Median | Batch2 Median | Change (%) |
| --- | --- | --- | --- | --- |
| R1 | Peak | 4.318 | 9.359 | 116.8 |
| L7 | Peak | 15.663 | 27.210 | 73.7 |
| R3 | Peak | 16.265 | 26.644 | 63.8 |
| L8 | Peak | 11.829 | 18.359 | 55.2 |
| R2 | Peak | 13.466 | 19.926 | 48.0 |
| L7 | Mean | 5.243 | 7.181 | 37.0 |
| R2 | Mean | 4.156 | 5.558 | 33.7 |
| L5 | Mean | 0.940 | 0.156 | -83.4 |
| L1 | Mean | 2.517 | 1.476 | -41.3 |
| L2 | Mean | 4.387 | 3.028 | -31.0 |

Note: Change = (Batch2 – Batch1) / Batch1 × 100%. Only sensors with |change| ≥ 30% are shown.

Table S6. Significant changes in IQR (|change| ≥ 50%)

| Sensor | Metric | Batch1 IQR | Batch2 IQR | Change (%) |
| --- | --- | --- | --- | --- |
| R1 | Peak | 3.202 | 8.790 | 174.5 |
| R3 | Peak | 7.249 | 15.885 | 119.1 |
| R2 | Peak | 6.277 | 11.124 | 77.2 |
| L3 | Peak | 6.199 | 10.889 | 75.6 |
| L7 | Peak | 5.932 | 9.672 | 63.1 |
| R5 | Mean | 0.769 | 0.235 | -69.5 |
| R5 | Peak | 1.211 | 0.574 | -52.6 |

Note: Change = (Batch2 – Batch1) / Batch1 × 100%. Only sensors with |change| ≥ 50% are shown.

Table S7. Comparison of global feature significance between 48‑subject and 80‑subject datasets

| Feature | 48 subjects p‑value |  | 80 subjects p‑value | Feature | 48 subjects p‑value | 80 subjects p‑value |  |  |  |
| --- | --- | --- | --- | --- | --- | --- | --- | --- | --- |
| Meanₓᴸ |  |  |  | TOTEXᵂ | +++ |  |  |  |  |
| Stdₓᴸ |  |  |  | CCAᵂ | +++ |  |  |  |  |
| Meanᵧᴸ |  |  |  | GA_Meanₓ |  |  |  |  |  |
| Stdᵧᴸ | +++ |  |  | GA_Stdₓ |  |  |  |  |  |
| MRDᴸ | ++ |  |  | GA_Meanᵧ |  |  |  |  |  |
| SRDᴸ | ++ |  | + | GA_Stdᵧ | + | + |  |  |  |
| TOTEXᴸ | +++ |  |  | SIM |  |  |  |  |  |
| CCAᴸ | +++ |  |  | JSD |  |  |  |  |  |
| Meanₓᴿ |  |  |  | GA_MRD |  |  |  |  |  |
| Stdₓᴿ | + |  |  | GA_SRD |  |  |  |  |  |
| Meanᵧᴿ |  |  |  | GA_TOTEX | ++ | ++ |  |  |  |
| Stdᵧᴿ | ++ |  |  | GA_CCA | + |  |  |  |  |
| MRDᴿ | + |  |  | GIC_Meanₓ |  |  |  |  |  |
| SRDᴿ | + |  | + | GIC_Stdₓ |  |  |  |  |  |
| TOTEXᴿ | +++ |  | + | GIC_Meanᵧ | +++ | ++ |  |  |  |
| CCAᴿ | ++ |  |  | GIC_Stdᵧ | ++ | ++ |  |  |  |
| Meanₓᵂ |  |  |  | SSIM |  |  |  |  |  |
| Stdₓᵂ |  |  |  | SJSD |  |  |  |  |  |
| Meanᵧᵂ |  |  |  | GIC_MRD |  | + |  |  |  |
| Stdᵧᵂ | +++ |  |  | GIC_SRD |  | + |  |  |  |
| MRDᵂ | +++ |  |  | GIC_TOTEX | + | ++ |  |  |  |
| SRDᵂ | ++ |  | ++ | GIC_CCA |  |  |  |  |  |

Note: +++ denotes $p<0.001$, ++ denotes $p<0.01$, + denotes $p<0.05$; blank cells indicate $p\geq0.05$ (not significant). The 48‑subject dataset corresponds to the original study, while the 80‑subject dataset is the merged dataset used in this study.

Table S8. Mapping of raw feature names to biomechanical labels used in SHAP analysis (Figures 5 and 6)

| Raw feature name | Biomechanical label (as shown in Figures 5 and 6) |
| --- | --- |
| **Global features (MLP1, Figure 5)** |  |
| gic_mean_y | Gait inconsistency of COP AP mean |
| totex_R | Right COP trajectory length |
| std_x_R | Right COP ML variability |
| gic_std_y | Gait inconsistency of COP AP variability |
| std_x_L | Left COP ML variability |
| srd_L | Left COP resultant distance variability |
| mean_x_W | Weak-foot COP ML mean |
| ga_totex | Gait asymmetry of COP trajectory length |
| mrd_L | Left COP mean resultant distance |
| std_y_R | Right COP AP variability |
| mrd_R | Right COP mean resultant distance |
| gic_std_x | Gait inconsistency of COP ML variability |
| mean_x_R | Right COP ML mean |
| srd_R | Right COP resultant distance variability |
| totex_W | Weak-foot COP trajectory length |
| gic_totex | Gait inconsistency of COP trajectory length |
| gic_srd | Gait inconsistency of COP resultant distance variability |
| gic_cca | Gait inconsistency of COP 95% circle area |
| cca_R | Right COP 95% confidence circle area |
| ga_std_y | Gait asymmetry of COP AP variability |
| **Local features (MLP2, Figure 6)** |  |
| Left_Transfer_Efficiency_Std | Variability of left pressure transfer efficiency |
| Left_Fore_Rear_Correlation_Std | Left forefoot-rearfoot coordination variability |
| Fore_Rear_Correlation_Mean_SI | Symmetry index of forefoot-rearfoot coordination |
| Transfer_Efficiency_Mean_SI | Symmetry index of pressure transfer efficiency |
| Right_R3_PTI_Mean | Mean PTI at right forefoot sensor R3 |
| Right_Fore_Rear_Correlation_Mean | Mean right forefoot-rearfoot coordination |
| Left_L4_FWHM_Mean | Mean pressure duration at left forefoot sensor L4 |
| Left_Toe_Peak_Pressure_Std | Variability of left toe peak pressure |
| Transfer_Efficiency_Mean_GA | Gait asymmetry of pressure transfer efficiency |
| Heel_Peak_Time_Mean_GA | Gait asymmetry of heel peak-pressure timing |
| Right_R3_FWHM_Mean | Mean pressure duration at right forefoot sensor R3 |
| Right_R3_AP_Mean | Mean pressure at right forefoot sensor R3 |
| Fore_Rear_Correlation_Mean_GA | Gait asymmetry of forefoot-rearfoot coordination |
| Left_L6_FWHM_Mean | Mean pressure duration at left midfoot sensor L6 |
| Left_L3_AP_Std | Variability of pressure at left forefoot sensor L3 |
| Left_Pressure_Transfer_Rate_Std | Variability of left pressure transfer rate |
| Left_Rearfoot_Load_Ratio_Integral_Std | Variability of left rearfoot load ratio |
| Right_Initial_To_Balance_Time_Std | Variability of right initial-to-balance time |
| Initial_To_Balance_Time_Mean_GA | Gait asymmetry of initial-to-balance time |
| Left_Forefoot_Load_Ratio_Integral_Std | Variability of left forefoot load ratio |

Notes: The biomechanical labels shown in Figures 5 and 6 are designed to be clinically interpretable; Readers may refer to the original feature definitions in the Methods section (Construction of the Local Feature Library and Global Feature Extraction) for detailed calculation formulas; COP = center of pressure; AP = anterior‑posterior; ML = medial‑lateral; PTI = pressure‑time integral.

Table S9. Fold-specific architecture and training hyperparameters of the four sub-models.

| Fold | Model | Architecture parameters | Learning rate | Dropout | Batch size |
| --- | --- | --- | --- | --- | --- |
| 1 | MLP1 | hidden dims = [44, 43] | 8.8095e-4 | 0.2246 | 16 |
| 1 | MLP2 | hidden dims = [16] | 5.0154e-4 | 0.2773 | 16 |
| 1 | CNN1 | conv channels = 24; blocks = 2; kernels = [5, 7]; FC units = 17 | 7.0229e-4 | 0.2840 | 16 |
| 1 | CNN2 | conv channels = 34; blocks = 2; kernels = [4, 8, 16]; FC units = 39 | 9.4954e-4 | 0.4976 | 16 |
| 2 | MLP1 | hidden dims = [55] | 8.3600e-4 | 0.4487 | 16 |
| 2 | MLP2 | hidden dims = [30] | 8.1039e-4 | 0.4565 | 32 |
| 2 | CNN1 | conv channels = 40; blocks = 2; kernels = [3, 5, 7, 9]; FC units = 23 | 9.8397e-4 | 0.4873 | 16 |
| 2 | CNN2 | conv channels = 57; blocks = 2; kernels = [4, 16]; FC units = 17 | 4.8085e-4 | 0.3801 | 16 |
| 3 | MLP1 | hidden dims = [42, 25] | 9.5741e-4 | 0.2736 | 32 |
| 3 | MLP2 | hidden dims = [27] | 7.4281e-4 | 0.2403 | 32 |
| 3 | CNN1 | conv channels = 34; blocks = 2; kernels = [4, 6, 8, 16]; FC units = 54 | 6.9220e-4 | 0.4120 | 64 |
| 3 | CNN2 | conv channels = 61; blocks = 2; kernels = [3, 5, 7, 9]; FC units = 33 | 9.8278e-4 | 0.4854 | 16 |
| 4 | MLP1 | hidden dims = [31] | 9.2518e-4 | 0.2194 | 32 |
| 4 | MLP2 | hidden dims = [21, 30] | 6.8185e-4 | 0.2304 | 16 |
| 4 | CNN1 | conv channels = 63; blocks = 1; kernels = [3, 5, 7, 9]; FC units = 54 | 2.2239e-4 | 0.4819 | 16 |
| 4 | CNN2 | conv channels = 34; blocks = 2; kernels = [3, 7]; FC units = 27 | 8.1442e-4 | 0.2327 | 16 |
| 5 | MLP1 | hidden dims = [59] | 9.0271e-4 | 0.4137 | 16 |
| 5 | MLP2 | hidden dims = [60, 64] | 5.7392e-4 | 0.3558 | 16 |
| 5 | CNN1 | conv channels = 54; blocks = 2; kernels = [4, 16]; FC units = 36 | 7.8805e-4 | 0.2189 | 16 |
| 5 | CNN2 | conv channels = 60; blocks = 2; kernels = [3, 7]; FC units = 34 | 9.5439e-4 | 0.4590 | 16 |
| 6 | MLP1 | hidden dims = [50, 35] | 7.4573e-4 | 0.3533 | 32 |
| 6 | MLP2 | hidden dims = [30, 47] | 6.7533e-4 | 0.3051 | 64 |
| 6 | CNN1 | conv channels = 55; blocks = 2; kernels = [4, 6, 8, 16]; FC units = 21 | 8.7970e-4 | 0.3611 | 64 |
| 6 | CNN2 | conv channels = 64; blocks = 2; kernels = [3, 5, 7, 9]; FC units = 47 | 9.5658e-4 | 0.3927 | 16 |
| 7 | MLP1 | hidden dims = [24] | 8.0259e-4 | 0.2649 | 16 |
| 7 | MLP2 | hidden dims = [41] | 7.0466e-4 | 0.2846 | 64 |
| 7 | CNN1 | conv channels = 63; blocks = 1; kernels = [4, 16]; FC units = 47 | 3.5146e-4 | 0.3713 | 16 |
| 7 | CNN2 | conv channels = 28; blocks = 2; kernels = [4, 8, 16]; FC units = 42 | 9.9433e-4 | 0.3582 | 16 |
| 8 | MLP1 | hidden dims = [64] | 9.4999e-4 | 0.3746 | 16 |
| 8 | MLP2 | hidden dims = [46] | 3.4994e-4 | 0.4968 | 32 |
| 8 | CNN1 | conv channels = 32; blocks = 2; kernels = [4, 16]; FC units = 33 | 4.5265e-4 | 0.3631 | 32 |
| 8 | CNN2 | conv channels = 28; blocks = 2; kernels = [4, 8, 16]; FC units = 32 | 6.1707e-4 | 0.3513 | 16 |

Note: MLP = multilayer perceptron; CNN = convolutional neural network; FC = fully connected layer; SE = squeeze-and-excitation. MLP1 and MLP2 used fully connected networks with ReLU activation and dropout after each hidden layer. CNN1 and CNN2 used multi-channel 1D convolutional networks with parallel kernel scales and an SE attention module. The learning rate, dropout rate, and batch size were fold-specific hyperparameters optimized within the inner cross-validation procedure. All models were trained for 100 epochs using the Adam optimizer and binary cross-entropy loss.

Fig.S1. Distribution of mean pressure across sensors for the two batches.

Fig.S2. Distribution of peak pressure across sensors for the two batches.

Fig.S3. Permutation test results for the distribution difference between two batches in the 44‑dimensional global feature space.

Fig.S4. Permutation test of PLS-DA classification performance between two batches in the 44-dimensional global feature space

Fig.S5. Model performance metrics on Batch1

Fig.S6. Model performance metrics on Batch2
